# Investigating Ganglion Cell Complex Thickness in Children with Chronic Heart Failure due to Dilated Cardiomyopathy

**DOI:** 10.3390/jcm9092882

**Published:** 2020-09-07

**Authors:** Klaudia Rakusiewicz, Krystyna Kanigowska, Wojciech Hautz, Lidia Ziółkowska

**Affiliations:** 1 Department of Ophthalmology, Children’s Memorial Health Institute, 04-730 Warsaw, Poland; k.kanigowska@ipczd.pl (K.K.); w.hautz@ipczd.pl (W.H.); 2 Department of Cardiology, Children’s Memorial Health Institute, 04-730 Warsaw, Poland; l.ziolkowska@ipczd.pl

**Keywords:** ganglion cell complex, retinal ganglion cells, optical coherence tomography, dilated cardiomyopathy, chronic heart failure

## Abstract

Purpose: To assess ganglion cell complex (GCC) thickness in children with chronic heart failure (CHF) due to dilated cardiomyopathy (DCM) using optical coherence tomography (OCT). Methods: Sixty eyes of 30 patients with chronic heart failure (CHF) due to dilated cardiomyopathy (DCM) and 60 eyes of 30 age- and sex-matched healthy volunteers (control group) were enrolled. The mean age of the patients and controls was 9.9 ± 3.57 (range 5–17) years and 10.08 ± 3.41 (range 4–16) years, respectively. All patients underwent a complete ophthalmic assessment and OCT imaging using RTVue XR Avanti (Optovue). The following OCT-based parameters were analysed: average ganglion cell complex thickness (avgGCC), superior ganglion cell complex thickness (supGCC), inferior ganglion cell complex thickness (infGCC), global loss of volume (GLV) and focal loss of volume (FLV). Results: There were no significant differences in avgGCC (98.13 μm vs. 99.96 μm, *p* = 0.21), supGCC (97.17 μm vs. 99.29 μm, *p* = 0.13), infGCC (99.03 μm vs. 100.71 μm, *p* = 0.25), FVL (0.49% vs. 0.4%, *p* = 0.25) and GVL (2.1% vs. 1.3%, *p* = 0.09) between patients with chronic heart failure due to dilated cardiomyopathy and healthy children. There was no correlation between avgGCC, supGCC, infGCC, FLV, GLV and ocular biometry, refractive errors or age. There was no correlation between avgGCC, supGCC, infGCC, FLV, GLV and NT-proBNP or LVEF. There were no significant differences in the studied parameters between the sexes. There were no significant differences in the studied parameters between the left and right eye. Conclusion: Our study seems to be the first to analyse ganglion cell complex in paediatric patients with dilated cardiomyopathy. We have demonstrated no changes in the ganglion cell complex thickness parameters in children with chronic heart failure due dilated cardiomyopathy, as compared to their healthy peers.

## 1. Introduction

The retinal ganglion cells (RGCs) are the sole output neurons responsible for the integration and transmission of all visual information from the retina to the brain [1]. Axons, cell bodies and dendrites of retinal ganglion cells located in three inner, separate layers of the retina form the retinal ganglion cell complex (GCC) [2,3].

The retinal ganglion cell complex thickness can be measured using a non-invasive, in vivo retinal imaging method: optical coherence tomography (OCT) [2,4,5]. OCT provides a high-resolution image of the retina and its individual anatomical layers and offers good repeatability. Computerised segmentation algorithms used on images obtained using OCT enable identification and thickness measurement of the three innermost retinal layers that compose the GCC: the ganglion cell layer (GCL), the inner plexiform layer (IPL) and the nerve fibre layer (NFL) [2,3,4,5,6]. As over 50% of the RGCs are located near the macula, the macular region makes a perfect target for detecting early changes in the RGC count, due to their high density [2,6].

The GCC parameters may serve as markers of diseases involving nerve tissue damage, and recently neurodegenerative diseases as well [2,7,8]. They have been known and widely used in glaucoma diagnosis and monitoring for many years [2,8]. Glaucoma research provided insight into the sequence of nerve cell damage, with the dendrite being damaged first, followed by the nerve cell body and the axon [6,8]. This sequence of retinal ganglion cell death implies that GCC changes first affect the inner plexiform layer (IPL), followed by the ganglion cell layer (GCL) and eventually the nerve fibre layer (NFL) [3,9]. Therefore, the GCC parameters are considered the most sensitive indicators of any adverse factors affecting the retina [10].

Retinal ganglion cells are known for their exceptional susceptibility to mild, transient and acute systemic hypoxic stress [10,11]. Hypoxia causes apoptosis of retinal ganglion cells. Reactive oxygen species produced under ischemic and hypoxic conditions disturb the balance between antioxidant and oxidant systems, resulting in ganglion cell death [11]. The inner retinal layers are more sensitive to hypoxia than the outer layers [6,10,11,12,13].

The retinal ganglion cell loss causing progressive damage to the optic nerve and visual impairment is a well-known phenomenon in such conditions as glaucoma, hereditary optic neuropathy, optic neuritis and ischemic optic neuropathy [14,15,16,17,18,19]. In systemic diseases, especially cardiovascular, systemic circulatory impairment leads to changes in retinal vascularisation [20,21,22,23,24]. Although the effect of retinal circulatory disturbance and impaired oxygen supply on retinal nerve cells, including the GCC complex, has not been thoroughly studied to date, it seems plausible and cannot be excluded.

Dilated cardiomyopathy (DCM) is defined as left ventricular (LV) dilatation and LV systolic dysfunction in the absence of abnormal loading conditions or coronary artery abnormalities sufficient to cause the abnormality [25,26]. It is the most common paediatric cardiomyopathy, with a reported annual incidence of 0.58–0.78 cases per 100,000 children [27,28]. Epidemiological studies have reported that most children with DCM present are under the age of one year and up to 93% of them have features of congestive heart failure [27]. DCM is a significant cause of heart failure and sudden cardiac death and is the most common indication for heart transplants in the paediatric population [29,30,31]. The main signs include left ventricular enlargement, dilatation and systolic dysfunction [26,32]. Chronic heart failure and a reduced left ventricular ejection fraction in patients with DCM lead to insufficient oxygen supply to tissues, including the retina [29,30,31].

The aim of the study was to assess GCC thickness in children with CHF secondary to DCM and in healthy children using the OCT. It aimed to determine whether changes to systemic circulation observed in chronic heart failure due to dilated cardiomyopathy affect GCC thickness.

## 2. Materials and Methods

This observational cross-sectional study was conducted at the Children’s Memorial Health Institute in Warsaw between February 2019 and March 2020. It adhered to the tenets of the Declaration of Helsinki and was approved by the Bioethics Committee of the Children’s Memorial Health Institute in Warsaw on 11 September 2019—No 33/KBE/2019. All subjects gave their informed consent for inclusion before they participated in the study. All participants above 16 years of age and legal guardians of those below 16 years of age were provided explanations as to the nature and possible consequences of the study and expressed their written, informed consent to participate in the study.

A total of 60 eyes of 30 children (16M/14F, mean age 9.9 years ± 3.57; range 5–17) with chronic heart failure (CHF) due to dilated cardiomyopathy (DCM) and treated in the Department of Cardiology at the Children’s Memorial Health Institute were enrolled. The study group inclusion criteria included confirmed CHF due to DCM lasting more than six months with a left ventricular ejection fraction (LVEF) ≤ 55%. The control group consisted of 60 eyes of 30 healthy children, without diagnosed heart failure or other systemic as well as ocular disease, matched for sex (16M/14F) and age (mean age 10.08 ± 3.41; range 4–16). The exclusion criteria in both groups included ocular diseases, such as hereditary retinal dystrophy, glaucoma, uveitis, vitreoretinal diseases; previous ocular trauma, retinal laser photocoagulation, eye surgery, significant refractive error (spherical refractive error > ±3 Dsph, cylindrical refractive error > ±3 Dcyl), other systemic comorbidities, such as diabetes mellitus, hypertension, kidney disease, neurological diseases or a history of prematurity. Additionally, patients reluctant to cooperate during assessments and eyes with low-quality scans were excluded.

Clinical parameters collected in patients with DCM included serum level of N-terminal (NT)-prohormone BNP (NT-proBNP), a biomarker of heart failure, and left ventricular ejection fraction (LVEF) measured using the Simpson method during the 2D transthoracic echocardiography. Each patient underwent a full ophthalmic assessment, including best-corrected visual acuity (BVCA) assessed with Snellen’s chart, anterior segment slit lamp biomicroscopy, fundus examination, ocular axial length measurement and cycloplegic (1% Tropicamide) refraction testing.

**Figure 1:**
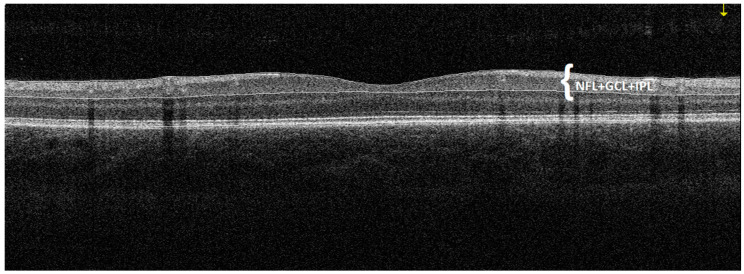
An example of a horizontal macular cross-section of a patient with DCM obtained by OCT. The GCC thickness is automatically measured by the device as the distance between the internal limiting membrane and the outer inner plexiform layer boundary. In the presented figure, three retinal layers such as the ganglion cell layer (GCL), the inner plexiform layer (IPL) and the nerve fibre layer (NFL), which together form a complex of retinal ganglion cells, are marked in white.

**Figure 2:**
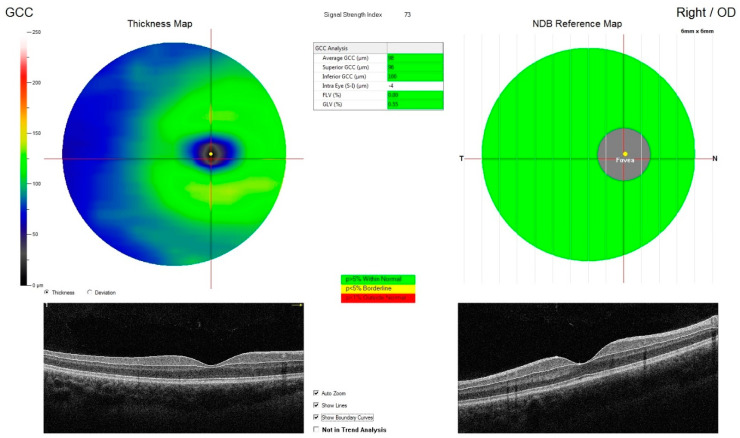
Measurement of retinal ganglion cell complex thickness in individual sectors of the eye such as the superior sector GCC thickness (supGCC), the inferior sector GCC thickness (infGCC) and the average thickness of both sectors (avgGCC).

The spectral domain OCT (SD-OCT) was performed in all participants using commercially available RTVue XR Avanti OCT system with AngioVue imaging system (Optovue, Fremont, CA, USA). The GCC scan consisting of a series of B-scans centred at 1 mm temporally to the fovea was taken in all cases. The GCC protocol consisted of 15 vertical lines with a 7 mm scanning length and a 0.5 mm interval, and one horizontal line with a 7 mm scanning length. Having analysed the scans, the device automatically calculated the GCC thickness defined as the distance between the internal limiting membrane (ILM) and the external boundary of the inner plexiform layer (IPL) (Figure 1). The RTVue XR Avanti device measures GCC separately for the superior and inferior eye sector, yielding three different values: the superior sector GCC thickness (supGCC), the inferior sector GCC thickness (infGCC) and the average thickness of both sectors (avgGCC) (Figure 2). Furthermore, the device automatically calculates two parameters: global loss volume (GLV) and focal loss volume (FLV). The GLV, which measures the average diffuse GCC loss across the entire scanned GCC area, is calculated from the fractional deviation map representing the percentage of GCC thickness reduction at each pixel location as compared to the normative database. The FLV, on the other hand, measures the average focal GCC loss across the entire scanned GCC area and is calculated by dividing the GCC thickness values at each location by the average GCC thickness across the entire map created for a given individual.

## 3. Statistical Analysis

The presented variables were expressed as means, standard deviations, 95% confidence intervals and ranges. The Wilcoxon two-way test for two independent samples (also known as the Mann–Whitney test) was used to determine the presence of statistical differences between the experimental group and the controls. It is a non-parametric alternative to Student’s t-test, which could not be used in the analysis due to the failure to meet the assumptions about the normal distribution of tested samples. Linear relationships between selected quantitative variables were calculated using the Pearson correlation coefficient of the product and angular momentum. The level *p* < 0.05 was considered statistically significant for all calculated comparisons. All statistical analyses were performed using R 3.5.1 software (R Core Team 2018).

## 4. Results

The data for 60 eyes of 30 patients with CHF in DCM (mean age 9.9 years ± 3.57) and 60 eyes of 30 healthy, sex- and age-matched controls (mean age 10.08 years ± 3.41) were included in the analyses. The mean NT-proBNP level in the study group was 568.1 pg/mL ± 1045.27 (normal value for the age of the studied patients < 125 ng/mL), while the mean LVEF was 49.03% ± 6.63 (normal value ≥ 55%). All participants had normal BCVA, as well as normal findings on anterior and posterior segment examination. The average axial eyeball length was 22.17 mm (± 0.88) in the DCM group and 22.46 mm (±0.65) in the control group (*p* = 0.11). The detailed characteristics of the entire study cohort are presented in Table 1.

**Figure 3:**
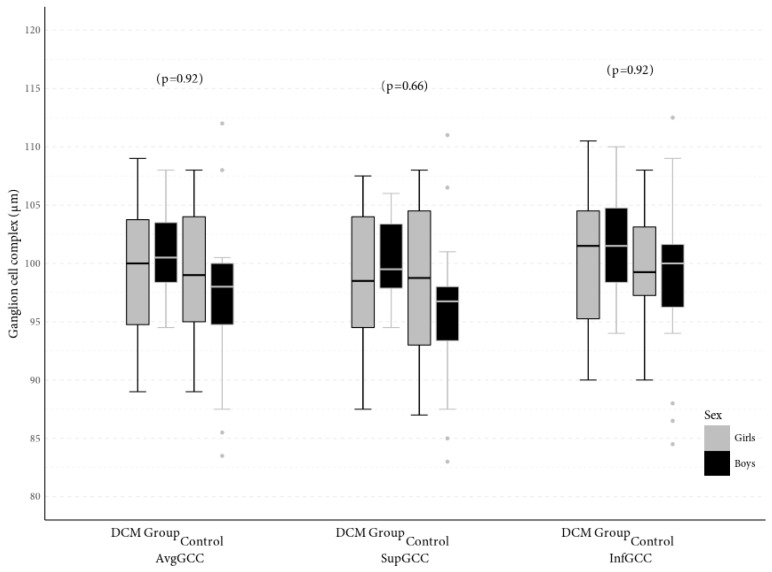
Central tendency and dispersion for GCC (μm) at selected anatomical locations in the study sample by DCM status and sex.

There were no significant differences in avgGCC (98.13 μm vs. 99.96 μm, *p* = 0.21), supGCC (97.17 μm vs. 99.29 μm, *p* = 0.13) or infGCC (99.03 μm vs. 100.71 μm, *p* = 0.25) between patients with chronic heart failure due to dilated cardiomyopathy and healthy children (Figure 3). Global loss volume (GLV) was 2.1% in the group of patients with DCM and 1.3% in the control group, while focal loss volume (FLV) was 0.49% in the children with DCM and 0.4% in the group of healthy children and was not statistically significantly different between groups (*p* = 0.09, *p* = 0.25, respectively).

**Figure 4:**
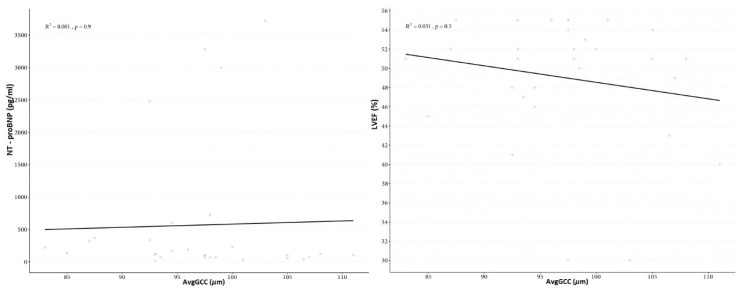
Graph of correlation between average ganglion cell complex thickness (avgGCC) and N-terminal (NT)-pro hormone BNP (NT-proBNP) and left ventricular ejection fraction (LVEF) in children with chronic heart failure in the course of dilated cardiomyopathy.

The means, standard deviations and ranges for the avgGCC, supGCC, infGCC, FLV and GLV in the study group and controls are presented in Table 2. There was no correlation between avgGCC, supGCC, infGCC, FLV, GLV and biometry, refractive errors or age. There was no correlation between avgGCC, supGCC, infGCC, FLV, GLV and NT-proBNP (*p* = 0.9, *p* = 0.8, *p* = 0.3, *p* = 0.8, *p* = 0.9, respectively). There was also no interconnection between avgGCC, supGCC, infGCC, FLV, GLV and LVEF (*p* = 0.3, *p* = 0.4, *p* = 0.8, *p* = 0.7, *p* = 0.5, respectively) (Figure 4). There were no significant differences in any studied parameters between the sexes.

## 5. Discussion

Dilated cardiomyopathy is a myocardial disease characterised by systolic dysfunction with concomitant tissue remodelling, which leads to heart failure [33]. It is the most common cardiomyopathy type in children [27,28]. Genetic, viral, immune, metabolic and cytotoxic factors have been implicated in the aetiology, although the cause remains unknown (idiopathic DCM) in almost 50% of cases [26]. In symptomatic patients, gradual disease progression is observed, with a mortality rate of approximately 30% within one to five years following the onset of clinical symptoms [28]. Left ventricular systolic dysfunction is the key pathophysiological aspect that plays a role in DCM development and progression [32]. As the left ventricle gets dilated, its walls get thinner. As a result, the most important myocardial function, that is, pumping blood to the systemic circulation and individual organs, becomes impaired. With the reduced left ventricular ejection fraction and arterial pressure, individual organs and tissues, including the eye and its structures, receive insufficient oxygen supply, which causes hypoxia [25,32].

Proper function of all retinal structures depends on regular and adequate oxygen supply [11]. The retina is known to be one of the most metabolically active tissues, so its oxygen demand is the highest of all tissues, about 10 times higher than that of the brain [34,35]. Retinal blood supply has two sources (dual/bipartite supply), which reflects the embryonic origin of the retina and ensures retinal oxygenation [36,37,38]. The outer retinal layers are supplied by choriocapillaries, whereas the inner retinal layers, including the retinal ganglion cells, are supplied by the capillaries originating from the central retinal artery that arises directly from the ocular artery [36,39]. The inner retinal layers are more susceptible to hypoxia, whereas the outer retinal layers are more resistant to hypoxic stress [6,10,11,12,13].

Choi et al. [40] observed that cerebral blood flow was 19% lower in 52 adult patients with advanced congestive heart failure secondary to idiopathic dilated cardiomyopathy than in healthy individuals. Experimental studies involving different models of retinal ischemia demonstrated RGC death in response to hypoxic-ischemic injury, triggered by a number of complex processes [41,42,43,44,45]. Retinal hypoxia upregulates the production of hypoxia-inducible factor-1α, vascular endothelial growth factor (VEGF), nitric oxide synthase (NOS), glutamate, inflammatory cytokines and reactive oxygen species (ROS), leading to cell apoptosis and tissue necrosis [11,44,45]. Furthermore, an association was confirmed between the retinal perfusion, RGC loss and retinal nervous activity under ischemic conditions [46,47].

Kergoat et al. [10] investigated the effect of breathing pure oxygen (O2), carbogen and a hypoxic gas on RGC function, which was measured using the pattern electroretinogram in 20 healthy men. They demonstrated that RGC function remained unchanged in response to increased blood oxygen and carbon dioxide levels, but it was changed in response to decreased blood oxygen levels, which indicates RGC sensitivity to transient, mild systemic hypoxia [10].

The majority of published studies carried out in patients with heart diseases, including heart failure, evaluated and analysed changes in the retinal nerve fibre layer (RNFL) only, not addressing GCC thickness changes [48,49,50]. Bayramoğlu et al. [51] assessed both retinal nerve fibre layer (RNFL) and GCC thickness using the OCT in 65 eyes in 33 adult patients with diastolic heart failure due to hypertrophic cardiomyopathy. They found no significant differences in the evaluated parameters between the study group and healthy controls. To the best of our knowledge, however, there are no studies to assess the GCC thickness in patients with CHF due to DCM, and there are definitely no such studies carried out in children, which makes our study innovative. Nevertheless, just as Bayramoğlu et al. [51], we did not observe significant differences in the GCC thickness between patients with CHF due to DCM and healthy children. Below we present a plausible explanation of this finding.

Glycolysis, angiogenesis, vasodilatation and erythropoiesis are autoregulatory, cellular and systemic response mechanisms which protect the human body in critical situations [11]. In heart failure, reduced cardiac output and hemodynamic imbalance trigger compensatory mechanisms, such as vasoconstriction, which aim at maintaining sufficient peripheral organ perfusion [50]. Consequently, the sympathetic nervous system and renin–angiotensin systems are activated [40]. Within the eye, systemic hypoxia has been reported to increase the diameter of retinal arteries and veins to ensure a stable blood flow [12,52,53]. However, the colour Doppler studies of the ophthalmic artery in patients with chronic heart failure demonstrated an increased vascular resistance index and a reduced flow rate [45]. The unique retinal survival capacity can also be linked to the ample presence of nutrients within the vitreous and the retina, such as glucose and glycogen, that get significantly depleted under hypoxia [54,55]. Even with a complete lack of oxygen, the retina can produce ATP in an anaerobic glycolysis cycle [56]. Animal studies demonstrated that the inner retinal layers, while more susceptible to oxidative stress, have better developed protective mechanisms against the effects of hypoxia [11,50]. An experimental study in cats demonstrated a significant reduction of oxygen partial pressure in the outer retinal layers in response to systemic hypoxia, while it remained unchanged in the inner retinal layers [37]. Those protective mechanisms may explain the absence of evident GCC damage in children with CHF due to DCM.

On the other hand, a number of studies confirmed the association between GCC thickness and parameters such as ocular axial length, patient age and sex [57,58,59,60,61,62]. In their study of 107 eyes, Zhao et al. [59] demonstrated GCC thinning in patients with greater axial eye length. This association between GCC thickness and ocular axial length was also confirmed by Takeyama et al. [58] as well as Sezgin Akcay et al. [60]. In their OCT-based study of 101 eyes, Hirasawa et al. [57] quantified this GCC thinning as 2.5 μm per each 1 mm increment of ocular axial length. In our study, we found no relationship between GCC thickness and ocular axial length in children with CHF due to DCM. This may be explained by a narrow age range of our cohort and exclusion of patients with high refractive errors, which significantly affect the ocular axial length.

Studies regarding the correlation between GCC and sex are inconclusive. Whereas there have been reports of the male sex being associated with higher GCC thickness [62,63], other studies disprove these conclusions [58,59]. We found no correlation between GCC thickness and sex in our study.

Most studies report a negative correlation between GCC thickness and age, indicating that GCC tends to be thinner in older patients [61,62,64,65]. Gao et al. [61] found RGCs to be the most vulnerable to age-related loss of all retinal cells, with the largest decline in their number between the second and fourth decade of life. Ooto et al. [62] analysed the thickness of individual retinal layers based on the OCT study in 256 healthy subjects, demonstrating a negative correlation between the age and the thickness of all three GCC-forming retinal layers, that is GCL, IPL and RNFL. The age-related GCL thinning was linear by 0.07 μm/year (0.2%/year). The same correlation was confirmed by Shariati et al. [64] in a murine model. In our study, we did not observe a correlation between GCC thickness and participant age. This can be explained by the young age of our cohort, with a narrow age range, as all above studies were conducted in adult patients. In line with the finding of GCC thinning from the second decade of life [61,62,64,65], it can be assumed that GCC thinning occurs later in the aging process and does not occur in childhood.

## 6. Conclusions

The retinal ganglion cell complex thickness was not reduced in children with chronic heart failure due to dilated cardiomyopathy as compared to their healthy peers. In our study group, no relationship was found between reduced systemic circulation associated with chronic heart failure and damage to retinal ganglion cells. Therefore, it can now be concluded that there is no clinical indication for standard evaluation of retinal ganglion cell complex in patients with dilated cardiomyopathy. Further longitudinal studies on a larger group of patients and with a longer follow- up of disease progression are needed to confirm the absence of changes to ganglion cell complex thickness in chronic heart failure in the paediatric population.

## Figures and Tables

**Table 1:** Study cohort characteristics.

Variable	Study Group	M	SD	95% CI	Range	*p*
NT-proBNP (pg/mL)	DCM Group	568.10	1045.27	177.79–958.41	15–3723	-
LVEF (%)	DCM Group	49.03	6.63	46.56–51.51	30–55	-
Age (years)	DCM Group	9.9	3.57	8.57–11.23	5–17	0.75
	Control group	10.08	3.41	8.97–11.18	4–16	
Biometry (mm)	DCM Group	22.17	0.88	21.85–22.5	20.615–24.08	0.11
	Control group	22.46	0.65	22.26–22.67	21.1–23.795	
	DCM Group	0.75	1.14	0.32–1.17	−2.00–3.00	0.47
spherical refractive error (Dsph)	Control group	0.56	1.17	0.19–0.94	−2.25–2.75	
	DCM Group	0.17	0.29	0.06–0.28	0.00–1.25	0.08
cylindrical eye error (Dcyl)	Control group	0.27	0.35	0.15–0.38	0.00–1.00	

M: mean; SD: standard deviation; CI: confidence interval; LVEF: left ventricular ejection fraction; NT-proBNP: natriuretic peptide type B.

**Table 2:** Descriptive statistics for GCC (μm) at selected anatomical locations (patients with DCM vs. controls).

Variable	Study Group	M	SD	95% CI	Range	*p*
avgGCC (μm)	DCM group	98.13	6.58	95.68–100.59	83.5–112	0.21
	Control group	99.96	5.25	98.26–101.66	89–109	
supGCC (μm)	DCM group	97.17	6.82	94.62–99.71	83–111	0.13
	Control group	99.29	5.28	97.58–101.01	87.5–107.5	
infGCC (μm)	DCM group	99.03	6.27	96.69–101.37	84.5–112.5	0.25
	Control group	100.71	5.51	98.92–102.49	90–110.5	
FLV (%)	DCM group	0.49	0.4	0.34–0.64	0.005–1.635	
	Control group	0.4	0.39	0.27–0.53	0.015–1.59	
GLV (%)	DCM group	2.1	2.73	1.08–3.12	0.005–11.5	0.09
	Control group	1.3	1.71	0.75–1.86	0.015–6.335	

M: mean; SD: standard deviation; CI: confidence interval; avgGCC: average ganglion cell complex; supGCC: superior ganglion cell complex; infGCC: inferior ganglion cell complex; FLV: focal loss volume; GLV: global loss volume.
